# Neighbourhood maternal socioeconomic status indicators and risk of congenital heart disease

**DOI:** 10.1186/s12884-020-03512-8

**Published:** 2021-01-21

**Authors:** Qun Miao, Sandra Dunn, Shi Wu Wen, Jane Lougheed, Jessica Reszel, Carolina Lavin Venegas, Mark Walker

**Affiliations:** 1 BORN Ontario, Ottawa, Ontario Canada; 2 Children’s Hospital of Eastern Ontario Research Institute, Ottawa, Ontario Canada; 3 School of Epidemiology and Public Health, University of Ottawa Faculty of Medicine, Ottawa, Canada; 4 OMNI Research Group, Clinical Epidemiology Program, Ottawa Hospital Research Institute, Ottawa, Canada; 5 School of Nursing, University of Ottawa, Ottawa, Ontario Canada; 6 Department of Obstetrics & Gynecology, University of Ottawa Faculty of Medicine, Ottawa, Canada; 7 Department of Pediatrics, Children’s Hospital of Eastern Ontario, Ottawa, Ontario Canada; 8 Department of Pediatrics, University of Ottawa, Ottawa, Ontario Canada

**Keywords:** The Better Outcomes Registry & Network (BORN) database, The Canadian Institute for Health Information Discharge Abstract Database (CIHI-DAD), Congenital heart disease, Socioeconomic status, Immigrants, Minorities

## Abstract

**Background:**

This study aimed to examine the relationships between various maternal socioeconomic status (SES) indicators and the risk of congenital heart disease (CHD).

**Methods:**

This was a population-based retrospective cohort study, including all singleton stillbirths and live births in Ontario hospitals from April 1, 2012 to March 31, 2018. Multivariable logistic regression models were performed to examine the relationships between maternal neighbourhood household income, poverty, education level, employment and unemployment status, immigration and minority status, and population density and the risk of CHD. All SES variables were estimated at a dissemination area level and categorized into quintiles. Adjustments were made for maternal age at birth, assisted reproductive technology, obesity, pre-existing maternal health conditions, substance use during pregnancy, rural or urban residence, and infant’s sex.

**Results:**

Of 804,292 singletons, 9731 (1.21%) infants with CHD were identified. Compared to infants whose mothers lived in the highest income neighbourhoods, infants whose mothers lived in the lowest income neighbourhoods had higher likelihood of developing CHD (adjusted OR: 1.29, 95% CI: 1.20–1.38). Compared to infants whose mothers lived in the neighbourhoods with the highest percentage of people with a university or higher degree, infants whose mothers lived in the neighbourhoods with the lowest percentage of people with university or higher degree had higher chance of CHD (adjusted OR: 1.34, 95% CI: 1.24–1.44). Compared to infants whose mothers lived in the neighbourhoods with the highest employment rate, the odds of infants whose mothers resided in areas with the lowest employment having CHD was 18% higher (adjusted OR: 1.18, 95% CI: 1.10–1.26). Compared to infants whose mothers lived in the neighbourhoods with the lowest proportion of immigrants or minorities, infants whose mothers resided in areas with the highest proportions of immigrants or minorities had 18% lower odds (adjusted OR: 0.82, 95% CI: 0.77–0.88) and 16% lower odds (adjusted OR: 0.84, 95% CI: 0.78–0.91) of CHD, respectively.

**Conclusion:**

Lower maternal neighbourhood household income, poverty, lower educational level and unemployment status had positive associations with CHD, highlighting a significant social inequity in Ontario. The findings of lower CHD risk in immigrant and minority neighbourhoods require further investigation.

## Background

Congenital heart disease (CHD) is a leading cause of infant morbidity and mortality in Canada and worldwide [1, 2]. The prevalence rate is estimated at 3.7 to 17.5 cases per 1000 live births, comprising 30 to 45% of all congenital anomalies (CAs) globally [1, 3–7]. In Canada, the overall CHD prevalence rate has been estimated at 12.3 per 1000 total births [8, 9]. In North America, it has been reported that 37% of deaths in infants with CAs are secondary to CHD [1, 3, 10].

The etiology of CHD remains unclear [3], although previous studies have reported that certain risk factors may contribute to the development of CHD, which include genetic factors and environmental factors such as advanced maternal age, rubella virus infection, exposure to environmental hazards during pregnancy, pre-pregnancy maternal obesity, the use of assisted reproductive technology (ART), certain medications, maternal social drug use, smoking, alcohol consumption during pregnancy, maternal pre-existing diabetes, and gestational diabetes [11–13].

Another potential risk factor for CHD is maternal socioeconomic status (SES) disparities [11, 12], although published findings regarding socioeconomic disparities and the risk of CHD are inconsistent. A recent meta-analysis combining data from 31 case-control studies and two cohort studies found that compared to reference groups, the risk of CHD was 11% higher for lower levels of maternal education (pooled RR = 1.11, 95% CI: 1.03, 1.21), 5% higher for lower family income (pooled RR = 1.05, 95% CI:1.01, 1.09), and 51% higher for maternal exposure to certain occupations (pooled RR = 1.51, 95% CI: 1.02, 2.24) [12]. However, the results were not consistent by all geographic areas and various SES indicators [12]. Conversely, another recent meta-analysis of two ecological, seven case-control and two cohort studies did not find associations between neighbourhood SES variables and the risk of CHD [13]. Moreover, a recent population-based study in Ontario, Canada found that children born in lower SES neighbourhoods (23% of all births) had 20% higher risk of CHD (RR: 1.20; 95% CI: 1.15–1.24) [14]. This finding is consistent with the results that we observed when we examined an association between ART and CHD in another study [15]. However, some studies had methodological limitations. For example, one meta-analysis that showed positive findings mainly relied on case-control studies with small sample sizes and may have been prone to information and selection bias [16]. Furthermore, a few studies conducted in Canada did not control for important confounders in their multivariable regression analyses [14, 17].

An explanation for this relationship between SES and risk of CHD remains unknown [12]. A lower SES level may be a proxy of environmental and behavioral factors such as smoking, social drug use, alcohol consumption, poor nutritional dietary habits, disadvantaged environmental living conditions, adverse maternal health conditions such as diabetes or uncontrolled residual confounding [12, 18, 19]. In addition, mothers with a lower SES level often experience poverty, which could lead to psychological stress, potentially elevating the production of corticosteroids, altering the immune system in pregnant women, and then possibly increasing the risk of having an infant with CHD [20, 21].

In Canada, social inequity has worsened, and income and wealth disparities have grown wider in the past two decades [22, 23]. According to 2016 Census data, 4.8 million Canadians are living in poverty and 1.2 million (20%) children live in low-income households [24]. It has been estimated that in Canada each year at least 50,000 children are born into poverty and 1 of 80–100 infants are born with CHD in Canada [4, 25]. Considering Ontario consists of 39% of Canada’s population, social inequity would be a major concern in prenatal health if pregnant women with a lower SES have an increased risk of CHD [26].

Moreover, SES has multiple dimensions; no single indicator can encompass all perspectives [27]. In the past, household income and education level were the two main SES factors studied [12]. There is limited research measuring other factors. In this study, we aimed to use multiple community SES factors to examine the relationships between SES and the risk of CHD from different angles.

## Methods

**Study design:** We conducted a population-based retrospective cohort study with Ontario data from April 1st 2012 to March 31st 2018.

### Study population

The study included all late stage terminations (pregnancies terminated at gestational age ≥ 20 weeks or birthweight ≥500 g) and births (live births and stillbirths) in Ontario hospitals from April 1st 2012 to March 31st 2018, with a birth weight ≥ 500 g, or gestational age ≥ 20 weeks. Records of mothers or infants residing outside of Ontario were excluded.

### Data sources

The Better Outcomes Registry & Network Ontario (BORN) is a registry that collects data on every pregnancy and birth in Ontario through the BORN Information System (BIS). The BORN prenatal databases capture data on maternal demographic characteristics and health behaviors, pre-existing maternal health problems, prenatal screening, obstetric complications, intra-partum interventions, fetal anomalies and outcomes in pregnancy, labour and birth, and postpartum stages [28]. The data are collected at various encounters but they are also aggregated into maternal pregnancy and infant datasets. Datasets in the BIS were used to perform the analysis including aggregate pregnancy, aggregate infant, antenatal specialty for high risk pregnant individuals, prenatal screening, and prenatal screening follow-up data. BORN strives to ensure high data quality in the BIS through an ongoing data validation process, quality checks, and formal training sessions for individuals entering data [28]. A number of papers and reports have been published using these data [28–31].

We also used the Discharge Abstract Database (DAD) and the National Ambulatory Care Reporting System (NACRS), which are run and maintained by the Canadian Institute for Health Information (CIHI) [32]. Each year, BORN receives CIHI-DAD and CIHI-NACRS data including maternal, newborn, and child (up to 1 year of age) records from acute care and emergency facilities in Ontario [33]. By using these data sources in conjunction with the BIS data, we are able to identify infants who had a diagnosis of CHD in hospital up to 1 year of age.

The 2016 Canadian Census and Postal Code Conversion File Plus (PCCF+) version 7B were employed for all neighbourhood data [34]. The 2016 Canadian Census is the latest Canada-wide census performed by Statistics Canada [35]. These data include Canadian social demographic information including household income, education, ethnicity, immigration, employment, and types of dwelling by different levels of geography [35]. Statistics Canada and Canada Post also developed the PCCF+, which contains postal codes matched with different levels of geography in Census data. The PCCF+ version 7B is the latest version reflecting the most up to date postal codes and their corresponding dissemination areas (DAs), which are small geographic areas, including 400–700 persons [34]. By linking these two files to the study cohort using maternal residence postal codes, we were able to obtain maternal neighbourhood level SES and minority and immigration status information at a dissemination area level.

### Data linkages

**Fig. 1:**
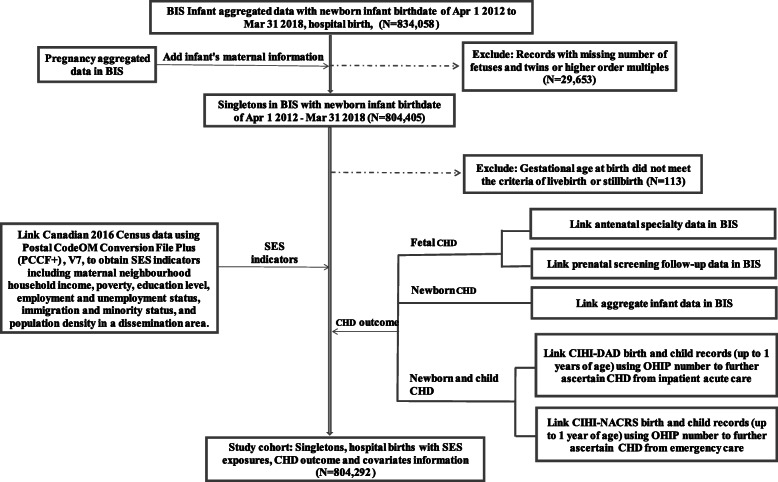
Flowchart of data sources and data linkage for the study cohort. Abbrev: BIS: BORN Information System, SES: socioeconomic status, OHIP: Ontario Health Insurance Plan, V7: version 7

The linkage process started within the BIS system. The baseline study cohort was obtained from the aggregate infant data of birthdates within the study timeframe. The outcome of CHD, SES variables (exposures) and covariates were obtained from multiple data sources (please see the data linkage flow chart in Fig. 1).

### Outcomes

All CHD cases captured in the prenatal stage were identified from the antenatal speciality and prenatal screening follow-up datasets in the BIS. Newborn diagnoses for CHD were collected from the birth child, postpartum child and neonatal intensive care encounters in the BIS and were aggregated into one infant dataset. We captured the newborn CHD from the aggregate infant dataset. Additional newborn CHD and CHD diagnosed during the infant’s first year were identified from the CIHI-DAD and CIHI-NACRS databases. In the BIS, CHD was coded in an anomaly picklist, which was based on clinical diagnosis. In the CIHI datasets, CHD was coded using the International Statistical Classification of Diseases and Related Health Problems, 10th Revision, Canadian adaptation (ICD-10-CA). Please see the CHD definitions of the BIS picklist values in the BIS data and the ICD-10-CA codes in the CIHI data in Appendix A.

### Covariates

This derived baseline infant dataset was linked to the aggregate maternal pregnancy data in the BIS to obtain maternal information including maternal age at delivery, conception type, pre-pregnancy body mass index (BMI), pre-pregnancy weight and height, pre-existing conditions and health conditions during pregnancy (including physical and mental health status), social drug use, as well as alcohol consumption and smoking status during pregnancy.

### SES measurement

SES, which reflects the social position or class of an individual, a family or a group of persons in a society, was linked to multiple social and economic factors [36]. There is no standard or universal SES measurement method in health equality research [36]. The most common indicators in the literature are household income and education [36]. Due to the multiple dimensions of SES, we included more indicators in this research. Based on the World Health Organization’s (WHO) [37] social determinants of health framework and available SES information, we measured SES using the indicators described below.

For the income related SES indicators, we assessed neighbourhood median household income after tax and before tax adjusted by size of household. Poverty was assessed according to the percentage of children aged 0–17 years living in low income households, the percentage of children aged 0–5 years living in low income households, and the percentage of all people with low income. Education level was assessed based on the percentage of adults aged 25–64 years having a university or higher degree and the percentage of adults aged 25–64 years without a high school diploma. We also assessed the percentage of minorities, the percentage of immigrants, the rate of unemployment among those aged 15 years or over, the rate of employment among those aged 15 years or over and population density. All SES and minority and immigration status indicators were assessed at a DA level in the province of Ontario and categorized as quintiles (Q1 = lowest, Q5 = highest) using the 2016 Canadian Census data.

### Statistical analysis

We first described the distributions of exposure variables and covariates by CHD. Spearman's rank-order correlations for ordinal variables were assessed among all SES variables. The Cochran-Armitage trend test was performed to evaluate the exposure of “dose-response” relationships between quintiles of all SES variables and the risk of CHD. Multivariable logistic regression models were conducted to examine the relationships between the maternal neighbourhood household income, poverty, education level, employment and unemployment status, immigration and minority status, and population density and the risk of CHD. Adjustments were made for maternal age at birth, ART, obesity, pre-existing health conditions, substance use during pregnancy, rural or urban residence, and infant’s sex. CHD is a rare outcome, therefore, odds ratios (ORs) and risk ratios are very close. Thus, we used odds ratios to estimate risk ratios.

Since most SES indicators were highly correlated, one SES variable was assessed in each separate multivariable regression model. All data linkages and analysis were performed using SAS 9.4 [38].

### Ethical considerations

This study was approved by the Children’s Hospital of Eastern Ontario Research Ethics Board and the Ottawa Health Science Network Research Ethics Board.

## Results

The study cohort consisted of 804,292 singletons born in hospital, including 3534 stillbirths (0.44% of the study population) and 1599 terminations (0.20% of the study population). In this cohort, 9731 (1.21%) infants with any type of CHD were identified, of which 300 fetuses (3.08% of 9731) were terminated and 125 fetuses (1.28% of 9731) were spontaneous losses. Table 1 shows the distributions of maternal and infant demographic factors and maternal behavioral factors, medical history, and health conditions. The average maternal age (mean ± SD) at birth was 30.56 ± 5.32 years. There were more male infants (412,809, 51.38% of total births) than female infants. The mean gestational age (mean ± SD) at birth was 38.79 ± 2.12 weeks. In total, 93,402 singletons (11.7%) lived in rural areas.

**Table 1:** Maternal and infant characteristics of study population

Variable*	Total cohort (***n*** = 804,292)
Maternal pre-pregnancy body mass index (BMI) in kg/m^2^	25.09 ± 6.09
Maternal age at birth in years, mean ± SD	30.56 ± 5.32
Obesity, BMI ≥ 30 kg/m^2^, yes,#(%)	122,494 (17.35)
ART conception, yes, #(%)	25,408 (3.16)
Maternal smoking or social drug use or alcohol consumption, yes, #(%)	96,313 (12.27)
Maternal smoking, yes, #(%)	79,502 (10.27)
Maternal alcohol consumption, yes,#(%)	17,652 (2.33)
Maternal social drug use, yes, #(%)	17,256 (2.27)
All types of mental health illness in pre-pregnancy or during pregnancy, yes, #(%)	119,522 (14.86)
Schizophrenia or bipolar diseases in pre-pregnancy or during pregnancy, yes, #(%)	4482 (0.56)
Depression in pre-pregnancy or during pregnancy, yes, #(%)	59,306 (7.37)
Anxiety in pre-pregnancy or during pregnancy, yes, #(%)	67,139 (8.35)
Pre_maternal health condition, yes, #(%)	149,593 (18.6)
Chronic hypertension, yes, #(%)	7464 (0.93)
Type I or type II diabetes, yes, #(%)	8769 (1.09)
History of heart disease, yes, #(%)	16,564 (2.06)
History of pulmonary disease, yes, #(%)	31,300 (3.89)
History of endocrine disease, yes, #(%)	39,792 (4.95)
Baby sex, male, #(%)	412,809 (51.38)
Baby GA at birth, mean ± SD	38.79 ± 2.12
Rural residence, yes, #(%)	93,402 (11.7)

*Missing values were excluded for % calculation

*Abbreviations*: *ART* assisted reproductive technology, *BMI* body mass index, *SD* standard deviation, *GA* gestational age

Table 2 shows the distributions of SES indicators categorized into quintiles on a DA level in Ontario. Compared to the distributions of median household income after tax adjusted by the size of household in the Ontario general population, there were more infants whose mothers lived in the poorest neighbourhoods (Q1, 24.01%) and fewer infants living in the richest neighbourhoods (Q5, 15.17%). The prevalence of CHD decreased from 1.33 (Q1) to 1.09 (Q5) accordingly, which was statistically significant based on the trend test (*p* < 0.0001). The prevalence and trend patterns were similar for other neighbourhood household income indicators, including median household income before tax adjusted by size of household, median household income after tax, and median household income before tax. For the variables of percentage of all people with low income and percentage of children aged 0–17 years with low income, the highest proportions of mothers (24.34 and 23.54%, respectively) lived in the poorest neighbourhood (Q5) and their infants had the highest prevalence of CHD (1.34 and 1.33%, respectively). For the variable children ages 0–5 years with low income, the highest proportion of mothers lived in the Q3 neighbourhoods (23.41%) and the highest CHD prevalence rate occurred in the Q5 neighbourhoods (1.40%). All three low income variables showed an increase in the prevalence of CHD rates from Q1 to Q5 with a statistically significant trend test.

**Table 2:** Distributions of SES, minority and immigration status indicators categorized into quintiles at a DA level in Ontario, Canada

Variable^a^	Total number	Percent (%)	Number of CHD	Percent of CHD (%)	p for trend*
Median household income after tax adjusted by size of household					< 0.0001
Missing	22,514	2.8	270	1.20	
Q1 (lowest)	193,099	24.01	2571	1.33	
Q2	161,321	20.06	2041	1.27	
Q3	158,058	19.65	1794	1.14	
Q4	147,314	18.32	1720	1.17	
Q5 (highest)	121,986	15.17	1335	1.09	
Median household income before tax adjusted by size of household					< 0.0001
Missing	22,514	2.8	270	1.20	
Q1 (lowest)	190,821	23.73	2565	1.34	
Q2	159,896	19.88	2034	1.27	
Q3	160,122	19.91	1815	1.13	
Q4	146,891	18.26	1712	1.17	
Q5 (highest)	124,048	15.42	1335	1.08	
Median household income after tax					< 0.0001
Missing	22,514	2.8	270	1.20	
Q1 (lowest)	173,182	21.53	2451	1.42	
Q2	155,121	19.29	1896	1.22	
Q3	153,183	19.05	1781	1.16	
Q4	164,328	20.43	1910	1.16	
Q5 (highest)	135,964	16.9	1423	1.05	
Median household income before tax					< 0.0001
Missing	22,514	2.8	270	1.20	
Q1 (lowest)	175,295	21.79	2495	1.42	
Q2	154,347	19.19	1871	1.21	
Q3	151,767	18.87	1805	1.19	
Q4	164,083	20.4	1860	1.13	
Q5(highest)	136,286	16.94	1430	1.05	
Percentage of children aged 0–17 with low income					< 0.0001
Missing	32,269	4.01	438	1.36	
Q1 (lowest)	129,282	16.07	1520	1.18	
Q2	151,539	18.84	1740	1.15	
Q3	151,448	18.83	1752	1.16	
Q4	150,422	18.7	1757	1.17	
Q5 (highest)	189,332	23.54	2524	1.33	
Percentage of children aged 0–5 with low income					< 0.0001
Missing	32,487	4.04	439	1.35	
Q1 (lowest)	154,439	19.2	1860	1.20	
Q2	131,720	16.38	1456	1.11	
Q3	188,274	23.41	2121	1.13	
Q4	140,413	17.46	1654	1.18	
Q5 (highest)	156,959	19.52	2201	1.40	
Percentage of all people with low income					< 0.0001
Missing	32,233	4.01	437	1.36	
Q1 (lowest)	123,415	15.34	1455	1.18	
Q2	152,352	18.94	1787	1.17	
Q3	153,204	19.05	1761	1.15	
Q4	147,328	18.32	1664	1.13	
Q5 (highest)	195,760	24.34	2627	1.34	
Percentage of people aged 25–64 without high school diploma					< 0.0001
Missing	22,206	2.76	264	1.19	
Q1 (lowest)	146,990	18.28	1533	1.04	
Q2	161,763	20.11	1908	1.18	
Q3	162,290	20.18	1999	1.23	
Q4	151,759	18.87	1905	1.26	
Q5 (highest)	159,284	19.8	2122	1.33	
Percentage of people aged 25–64 had a degree of university or higher					< 0.0001
Missing	22,206	2.76	264	1.19	
Q1 (lowest)	129,480	16.1	1862	1.44	
Q2	135,950	16.9	1773	1.30	
Q3	159,186	19.79	2009	1.26	
Q4	188,683	23.46	2129	1.13	
Q5 (highest)	168,787	20.99	1694	1.00	
Percentage of immigrants					< 0.0001
Missing	22,206	2.76	264	1.19	
Q1 (lowest)	121,601	15.12	1744	1.43	
Q2	115,179	14.32	1503	1.30	
Q3	131,853	16.39	1695	1.29	
Q4	174,205	21.66	1981	1.14	
Q5 (highest)	239,248	29.75	2544	1.06	
Percentage of minority					< 0.0001
Missing	22,206	2.76	264	1.19	
Q1 (lowest)	109,379	13.6	1539	1.41	
Q2	115,798	14.4	1607	1.39	
Q3	128,601	15.99	1617	1.26	
Q4	170,814	21.24	1864	1.09	
Q5 (highest)	257,494	32.01	2840	1.10	
Percentage of unemployment among aged 15 or over					< 0.0001
Missing	22,206	2.76	264	1.19	
Q1 (lowest)	125,616	15.62	1472	1.17	
Q2	167,581	20.84	1884	1.12	
Q3	170,043	21.14	2031	1.19	
Q4	159,195	19.79	1948	1.22	
Q5 (highest)	159,651	19.85	2132	1.34	
Percentage of employment among aged 15 or over					< 0.0001
Missing	22,206	2.76	264	1.19	
Q1 (lowest)	136,282	16.94	1892	1.39	
Q2	139,017	17.28	1710	1.23	
Q3	153,922	19.14	1786	1.16	
Q4	155,063	19.28	1884	1.21	
Q5 (highest)	197,802	24.59	2195	1.11	
Population density per kilometer					< 0.0001
Missing	21,548	2.68	246	1.14	
Q1 (lowest)	108,132	13.44	1413	1.31	
Q2	167,664	20.85	2083	1.24	
Q3	134,702	16.75	1685	1.25	
Q4	158,626	19.72	1958	1.23	
Q5 (highest)	213,620	26.56	2346	1.10	

^a^All variables were derived from Census 2016

*: *p* value for trend test was calculated after missing values were removed

This cohort was almost equally distributed within quintiles on the education variable of percentage of people aged 25–64 years without a high school diploma, while the CHD prevalence rate increased from Q1 to Q5 and showed a statistically significant trend. On the other hand, for the variable people aged 25–64 years who had a university degree or higher, the highest proportion of the study cohort was in Q4 (23.46%) ; the CHD prevalence rate decreased from Q1 to Q5 and showed a statistically significant trend. Around one-third of the birth cohort (29.75 and 32.01%, respectively) lived in neighbourhoods with the highest proportion of immigrants and minorities; for both variables, the CHD prevalence rate decreased from Q1 to Q5 and showed a statistically significant trend. This birth cohort showed that less than 20% of mothers lived in the neighbourhoods with the lowest unemployment rate, lowest employment rate and with the lowest population density. The prevalence of CHD showed an increasing trend with increasing unemployment rate and decreasing employment rate and population density.

All SES indicators show different degrees of correlations with statistical significance (Table 3). The Spearman's rank-order correlation coefficient between median household income after tax and before tax was close to one (0.98), and when adjusted by household size it was also close to one (0.98). The correlations among the percentage of all people with low income, percentage of children aged 0–17 years with low income, and percentage of children aged 0–5 years with low income was between 0.88 and 0.75. The correlation between the percentage of people aged 25–64 years who had a university degree or higher and without high school diploma was − 0.64. The percentage of employment among people aged 15 years or over and the percentage of unemployment among the same age group was − 0.48. The correlation between the percentage of minorities and the percentage of immigrants was 0.89. The population density per kilometer variable had a lower correlation (0.01 to 0.34) with other SES indicators except with the percentage of minorities (0.59) and the percentage of immigrants (0.57).

**Table 3:** Correlations between different SES, minority and immigration status indicators

	Spearman's rank-order correlation coefficient^a^ ***p*** value													
	Median household income after tax	Median household income before tax	Household Median income after tax adjusted by household size	Median household income before tax adjusted by household size	Percentage of all people with low income	Percentage of children aged 0-17 with low income	Percentage of children aged 0-5 with low income	Percentage of people aged 25-64 without a high school diploma	Percentage of people aged 25-64 with a university degree or higher	Percentage of unemployment among aged 15 or over	Percentage of employment among aged 15 or over	Percentage of minorities	Percentage of Immigrant	Population density per kilometer
**Median household income after tax**	1	0.98	0.68	0.71	-0.79	-0.72	-0.63	-0.48	0.40	-0.38	0.53	0.04	0.05	-0.20
		<.0001	<.0001	<.0001	<.0001	<.0001	<.0001	<.0001	<.0001	<.0001	<.0001	<.0001	<.0001	<.0001
**Median household income before tax**	0.98	1	0.71	0.74	-0.80	-0.73	-0.64	-0.51	0.42	-0.39	0.54	0.02	0.04	-0.20
	<.0001		<.0001	<.0001	<.0001	<.0001	<.0001	<.0001	<.0001	<.0001	<.0001	<.0001	<.0001	<.0001
**Median household income after tax adjusted by household size**	0.68	0.71	1	0.98	-0.73	-0.70	-0.64	-0.64	0.39	-0.45	0.46	-0.30	-0.27	-0.28
	<.0001	<.0001		<.0001	<.0001	<.0001	<.0001	<.0001	<.0001	<.0001	<.0001	<.0001	<.0001	<.0001
**Median household income before tax adjusted by household size**	0.71	0.74	0.98	1	-0.74	-0.71	-0.65	-0.64	0.40	-0.46	0.48	-0.28	-0.25	-0.28
	<.0001	<.0001	<.0001		<.0001	<.0001	<.0001	<.0001	<.0001	<.0001	<.0001	<.0001	<.0001	<.0001
**Percentage of all people with low income**	-0.79	-0.80	-0.73	-0.74	1	0.88	0.75	0.42	-0.16	0.44	-0.46	0.27	0.24	0.34
	<.0001	<.0001	<.0001	<.0001		<.0001	<.0001	<.0001	<.0001	<.0001	<.0001	<.0001	<.0001	<.0001
**Percentage of children aged 0-17 with low income**	-0.72	-0.73	-0.70	-0.71	0.88	1	0.79	0.42	-0.19	0.44	-0.45	0.29	0.25	0.33
	<.0001	<.0001	<.0001	<.0001	<.0001		<.0001	<.0001	<.0001	<.0001	<.0001	<.0001	<.0001	<.0001
**Percentage of children aged 0-5 with low income**	-0.63	-0.64	-0.64	-0.65	0.75	0.79	1	0.41	-0.22	0.40	-0.41	0.23	0.19	0.26
	<.0001	<.0001	<.0001	<.0001	<.0001	<.0001		<.0001	<.0001	<.0001	<.0001	<.0001	<.0001	<.0001
**Percentage of people aged 25-64 without a high school diploma**	-0.48	-0.51	-0.64	-0.64	0.42	0.42	0.41	1	-0.64	0.27	-0.39	-0.03	-0.03	0.01
	<.0001	<.0001	<.0001	<.0001	<.0001	<.0001	<.0001		<.0001	<.0001	<.0001	<.0001	<.0001	<.0001
**Percentage of people aged 25-64 with a university degree or higher**	0.40	0.42	0.39	0.40	-0.16	-0.19	-0.22	-0.64	1	-0.10	0.28	0.47	0.48	0.31
	<.0001	<.0001	<.0001	<.0001	<.0001	<.0001	<.0001	<.0001		<.0001	<.0001	<.0001	<.0001	<.0001
**Percentage of unemployment among aged 15 or older**	-0.38	-0.39	-0.45	-0.46	0.44	0.44	0.40	0.27	-0.10	1	-0.48	0.25	0.21	0.25
	<.0001	<.0001	<.0001	<.0001	<.0001	<.0001	<.0001	<.0001	<.0001		<.0001	<.0001	<.0001	<.0001
**Percentage of employment among aged 15 or older**	0.53	0.54	0.46	0.48	-0.46	-0.45	-0.41	-0.39	0.28	-0.48	1	-0.01	-0.06	-0.07
	<.0001	<.0001	<.0001	<.0001	<.0001	<.0001	<.0001	<.0001	<.0001	<.0001		<.0001	<.0001	<.0001
**Percentage of minorities**	0.04	0.02	-0.30	-0.28	0.27	0.29	0.23	-0.03	0.47	0.25	-0.01	1	0.89	0.59
	<.0001	<.0001	<.0001	<.0001	<.0001	<.0001	<.0001	<.0001	<.0001	<.0001	<.0001		<.0001	<.0001
**Percentage of Immigrants**	0.05	0.04	-0.27	-0.25	0.24	0.25	0.19	-0.03	0.48	0.21	-0.06	0.89	1	0.57
	<.0001	<.0001	<.0001	<.0001	<.0001	<.0001	<.0001	<.0001	<.0001	<.0001	<.0001	<.0001		<.0001
**Population density per kilometer**	-0.20	-0.20	-0.28	-0.28	0.34	0.33	0.26	0.01	0.31	0.25	-0.07	0.59	0.57	1
	<.0001	<.0001	<.0001	<.0001	<.0001	<.0001	<.0001	<.0001	<.0001	<.0001	<.0001	<.0001	<.0001	

^a^In total, 4% of data was missing and excluded from this analysis

Table 4 shows both crude ORs and ORs adjusted by covariates. Compared to infants whose mothers lived in Q5 neighbourhoods (highest income), infants whose mothers lived in Q1 neighbourhoods (lowest median household income after tax) had higher likelihood of developing CHD (adjusted OR: 1.29, 95% CI: 1.20–1.38). Compared to infants whose mothers lived in Q5 neighbourhoods (highest percentage of people with university or higher degrees among aged 25–64 population), infants whose mothers lived in Q1 neighbourhoods had higher odds of CHD (adjusted OR: 1.34, 95% CI: 1.24–1.44). Compared to infants whose mothers lived in the neighbourhoods with the highest employment rate, infants whose mothers resided in areas with the lowest employment rate had a higher likelihood of developing CHD (adjusted OR: 1.18, 95% CI: 1.10–1.26). Compared to infants whose mothers lived in the neighbourhoods with the lowest proportion of immigrants or minorities (Q1), infants’ whose mothers resided in areas with the highest proportions of immigrants or minorities (Q5) had a lower likelihood of developing CHD; specifically, there was 18% lower odds in Q5 immigrant neighbourhoods (adjusted OR: 0.82, 95% CI: 0.77–0.88) and there was 16% lower odds in Q5 minority neighbourhoods (adjusted OR: 0.84, 95% CI: 0.78–0.91). There was no significant difference in the risk of CHD for infants by population density of maternal residence.

**Table 4:** Associations between different maternal SES, minority and immigration status indicators and CHD

Variable	Crude OR	Adjust OR
Median household income after tax		
Q1 (lowest)	1.34 (1.26–1.44)	1.29 (1.20–1.38)
Q2	1.18 (1.1–1.26)	1.13 (1.05–1.22)
Q3	1.11 (1.03–1.19)	1.08 (1–1.16)
Q4	1.10 (1.03–1.19)	1.10 (1.02–1.18)
Q5 (highest)	Reference	Reference
Median household income before tax		
Q1 (lowest)	1.35 (1.26–1.44)	1.30 (1.21–1.39)
Q2	1.16 (1.08–1.25)	1.13 (1.05–1.21)
Q3	1.13 (1.06–1.22)	1.11 (1.03–1.19)
Q4	1.07 (1.00–1.15)	1.07 (1–1.15)
Q5 (highest)	Reference	Reference
Median household income after tax adjusted by size of household		
Q1 (lowest)	1.17 (1.09–1.25)	1.18 (1.10–1.27)
Q2	1.14 (1.06–1.23)	1.14 (1.06–1.23)
Q3	1.03 (0.95–1.1)	1.02 (0.95–1.1)
Q4	1.05 (0.98–1.13)	1.05 (0.97–1.13)
Q5 (highest)	Reference	Reference
Median household income before tax adjusted by size of household		
Q1 (lowest)	1.20 (1.12–1.29)	1.22 (1.13–1.30)
Q2	1.17 (1.09–1.26)	1.17 (1.09–1.26)
Q3	1.04 (0.97–1.12)	1.04 (0.96–1.12)
Q4	1.07 (1.00–1.16)	1.07 (1.00–1.15)
Q5 (highest)	Reference	Reference
Percentage of all people with low income		
Q1 (lowest)	Reference	Reference
Q2	1.00 (0.93–1.07)	1.00 (0.94–1.08)
Q3	0.96 (0.89–1.03)	0.97 (0.91–1.05)
Q4	0.96 (0.89–1.03)	0.96 (0.9–1.04)
Q5 (highest)	1.13 (1.06–1.21)	1.13 (1.06–1.21)
Percentage of children aged 0–17 with low income		
Q1 (lowest)	Reference	Reference
Q2	0.98 (0.91–1.05)	0.99 (0.93–1.07)
Q3	0.98 (0.91–1.05)	1.01 (0.94–1.08)
Q4	0.99 (0.93–1.07)	1.01 (0.94–1.08)
Q5 (highest)	1.14 (1.06–1.21)	1.14 (1.07–1.22)
Percentage of children aged 0–5 with low income		
Q1 (lowest)	Reference	Reference
Q2	0.92 (0.86–0.99)	0.95 (0.89–1.02)
Q3	0.93 (0.87–0.99)	0.96 (0.90–1.02)
Q4	0.97 (0.9–1.04)	0.99 (0.92–1.06)
Q5 (highest)	1.16 (1.09–1.24)	1.17 (1.10–1.25)
Percentage of people aged 25–64 with a university degree or higher		
Q1 (lowest)	1.42 (1.32–1.52)	1.34 (1.24–1.44)
Q2	1.30 (1.21–1.4)	1.26 (1.17–1.35)
Q3	1.25 (1.17–1.34)	1.24 (1.16–1.33)
Q4	1.11 (1.04–1.18)	1.11 (1.04–1.19)
Q5 (highest)	Reference	Reference
Percentage of people aged 25–64 without a high school diploma		
Q1 (lowest)	Reference	Reference
Q2	1.10 (1.03–1.18)	1.10 (1.02–1.18)
Q3	1.16 (1.08–1.24)	1.14 (1.07–1.23)
Q4	1.18 (1.1–1.27)	1.16 (1.08–1.25)
Q5 (highest)	1.24 (1.16–1.33)	1.21 (1.13–1.3)
Percentage of unemployment among aged 15 or over		
Q1 (lowest)	Reference	Reference
Q2	0.95 (0.89–1.02)	0.96 (0.90–1.03)
Q3	1.00 (0.93–1.07)	1.02 (0.95–1.09)
Q4	1.03 (0.96–1.1)	1.05 (0.98–1.12)
Q5 (highest)	1.09 (1.02–1.17)	1.09 (1.02–1.17)
Percentage of employment among aged 15 or over		
Q1 (lowest)	1.22 (1.15–1.30)	1.18 (1.10–1.26)
Q2	1.11 (1.04–1.18)	1.09 (1.02–1.16)
Q3	1.05 (0.98–1.12)	1.04 (0.98–1.11)
Q4	1.10 (1.03–1.17)	1.09 (1.02–1.16)
Q5(highest)	Reference	Reference
Percentage of minorities		
Q1 (lowest)	Reference	Reference
Q2	1.01 (0.94–1.09)	0.99 (0.92–1.07)
Q3	0.92 (0.85–0.99)	0.90 (0.83–0.98)
Q4	0.79 (0.74–0.85)	0.80 (0.74–0.87)
Q5 (highest)	0.78 (0.73–0.84)	0.84 (0.78–0.91)
Percentage of immigrants		
Q1 (lowest)	Reference	Reference
Q2	0.93 (0.87–1.00)	0.94 (0.88–1.01)
Q3	0.92 (0.86–0.99)	0.94 (0.87–1.01)
Q4	0.81 (0.76–0.87)	0.86 (0.79–0.92)
Q5 (highest)	0.74 (0.69–0.79)	0.82 (0.77–0.88)
Population density per kilometer		
Q1 (lowest)	Reference	Reference
Q2	0.98 (0.91–1.05)	1.05 (0.94–1.18)
Q3	0.98 (0.91–1.06)	1.05 (0.93–1.18)
Q4	0.97 (0.9–1.04)	1.05 (0.93–1.19)
Q5 (highest)	0.86 (0.8–0.92)	0.96 (0.85–1.08)

Note. Multivariable logistic regression models were performed to calculate adjusted ORs. Adjusted ORs for each SES variable were in one separate model adjusting for covariates including maternal age at birth, pre-pregnancy obesity, maternal smoking or social drug use or alcohol consumption, mental health illness in pre-pregnancy or during pregnancy, pre-pregnancy maternal health condition, infant sex and maternal residence in rural area

## Discussion

In our study of 804,292 singletons born in hospital, 9,731 (1.21%) infants with CHD were identified. After adjusting for potential confounders including maternal age, maternal behavior factors, medical conditions, infant sex, and rural or urban maternal residence, we still found that certain SES variables increased the risk of CHD, specifically those related to income, education, and employment. Compared to infants whose mothers lived in the communities with the lowest percentage of immigrants or minorities, infants had lower odds of CHD if the infants’ mothers lived in the communities with the highest percentage of immigrants or minorities. No association was found between population density of maternal residence and risk of CHD. In this study, late stage terminations and births (live births and stillbirths) were combined in the analysis. Given that pregnancy outcome could be a marker of severity of CHD, we conducted a sensitivity analysis by excluding both stillbirths and terminations, as well as by excluding terminations only from the cohort. The results are very similar (Appendix B).

This large population-based birth cohort represents all singleton hospital births in Ontario from the fiscal years of 2012 (April 1, 2012 – March 31, 2013) to 2017 (April 1, 2017 – March 31, 2018). We identified prenatal and postnatal CHD cases (up to the first year of life) by linking multiple databases. The overall CHD prevalence rate (1.21 per 100 births) that we observed was consistent with that reported in the literature in Canada and worldwide [1, 3–6]. A study using national CIHI data (excluding the province of Quebec) reported that the CHD prevalence between 1990 and 2011 was 12.3 per 1000 live births and stillbirths [8, 9]. Another study that used CIHI data between 1994 and 2007 found a prevalence rate of 15.1 per 1000 live births [14].

In our study, we examined the relationships between different community median household income-related SES variables and the risk of CHD. Compared to infants whose mothers lived in Q5 neighbourhoods, the odds of having a CHD among infants whose mothers resided in Q1, Q2, Q3 and Q4 were similar using the variables of ‘median household income after tax’ or ‘median household income before tax’. The median household income after tax variable should be more accurate than the one before tax because the former reflects the actual income that a household receives. However, we used quintiles of household income at a DA level, thereby minimizing the actual difference. In terms of median household income or median household income adjusted by size of household, the ORs of CHD among infants was higher for Q1 neighbourhoods compared to Q5 neighbourhoods (OR: 1.29, 1.30, 1.18 and 1.22 for median household income after tax, median household income before tax, median household income after tax adjusted by size of household and median household income before tax adjusted by size of household, respectively). The ORs for the other categories (Q2 vs. Q5, Q3 vs. Q5, and Q4 vs. Q5) were similar for these four variables. Median household income unadjusted by household size is more frequently used in research. There was no consensus if we should have used median household income or median household income adjusted by household size, as none of them capture the actual financial condition of the household. Through examining these four indicators, we concluded that the most significant effect on the risk of CHD occurred in the least wealthy areas. This finding is consistent with that in the literature [12].

In terms of the three poverty indicators (percentage of all people with low income, percentage of children aged 0–17 years with low income and percentage of children aged 0–5 years with low income), they all showed a similar pattern. Infants whose mother resided in an area with the highest percentage of people with low income (Q5) had a higher risk of CHD when compared to infants whose mother lived in an area with the lowest percentage of people with low income (Q1).

All household income variables and poverty indicators are related with families’ material resources. A family with a lower income may only be able to afford to live in an area with disadvantaged living environments such as high pollution and less green space [36]. Rented homes may be associated with poor indoor air quality [36]. Furthermore, lower income families may only be able to afford lower quality food. Poorer financial conditions may increase stress levels as well, which could increase the risk of CHD [20, 21].

Regarding education level indicators (percentage of people aged 25–64 years without a high school diploma and percentage of people aged 25–64 years with a university degree or higher), there was a higher risk of CHD among infants when the education level of those in the household was lower. Lower education level may be a proxy of lack of knowledge on prevention of adverse maternal and birth outcomes including healthy diet and physical activity etc., which we were not able to measure in the analysis due to data limitations. In addition, a lower education level tends to be correlated with a lower income [27]. Thus, any mechanisms through which lower family income lead to a higher risk of CHD may also apply to education indicators.

Unemployment and employment rates also show a similar pattern with household income and education indicators. Employment or unemployment status influences a household’s income, which may be directly related to a family’s purchasing power for housing with higher quality living conditions, healthy foods and maintaining healthy behaviors [27, 39].

Minority and immigrant groups are often characterized as socially disadvantaged groups because most of them, especially newcomers, likely lack social and financial support and have underdeveloped social connections [39]. This could increase maternal stress levels and thus potentially elevate the risk of adverse birth outcomes [20, 21, 36]. However, in our study, the findings of minority and immigrant indicators on the risk of CHD are in the opposite direction than expected. One recent published study also found similar results using Ontario hospital births and refugee/immigrant population-based data between April 1, 2002 and March 31, 2014 [40]. The study showed that compared to Canadian-born pregnant women, non-refugee immigrants (adjusted OR: 0.86, 95% CI: 0.84–0.88) and refugee immigrants (adjusted OR: 0.87, 95% CI: 0.83–0.91) had lower odds of having a child with congenital anomalies [40]. The unexpected finding may be due to the healthy immigrant effect [41]. In Canada, many minorities are recent immigrants [29]. A number of studies have shown that the health of recently immigrated women is better than the health of Canadian-born women and the women in the original countries where these immigrants are from [41]. This healthy immigrant effect may be due to the Canadian government’s selection of healthy immigrants and immigrants’ “self-selection of healthier individuals” from their mother countries [41]. In addition, new immigrants may tend to have healthier behaviors including no smoking, less alcohol and drug consumption, physical activity and healthy diets [41, 42]. To further investigate the relationships between immigrants and minorities and the risk of CHD in infants, an individual-level analysis is needed as the indicators of minorities and immigrants at a community level may not represent the true relationships with the risk of CHD [43].

This study had several strengths. We included all singleton births from fiscal year 2012–13 to fiscal year 2017–18 in the province of Ontario, Canada. The large sample size improved the precision of the study results. The prevalence of CHD represented the target population of Ontario. Furthermore, the CHD cases were ascertained by linking multiple data sources including those identified in prenatal, postnatal or birth records and those identified up to 1 year of infancy. Many other studies using birth registry data lacked sufficient resources to conduct analysis on the complete data.

There were a few limitations in this study. First, due to data limitations, we were not able to use the individual family’s household income, the mother’s education level and employment status to evaluate the relationships between the maternal SES factors and the risk of CHD among infants. Instead, we had to rely on information from the neighbourhoods in which mothers resided. However, in Canada, neighbourhood SES factors are based on the DA level (a small geographic area, including 400–700 persons) and have been considered as good proxies of individual-level maternal SES factors as reported in previous studies [44]. The findings of our study are consistent with that from studies using individual-level SES indicators[12, 45, 46]. For example, one case-control study conducted in Lithuania found that compared to mothers who received advanced vocational training or higher education, those mothers who received low and moderate education levels were 3.4 times more likely to have a child with CHD [45]. Another study conducted in China found low household income and mothers without a high school diploma were associated with certain types of CHD, including ventricular septal defect, atrial septal defect and pulmonary stenosis [46].

In addition, neighbourhood SES variables may also reflect the conditions of external environmental factors and accessibility to social and health services that might influence the prevalence of CHD. For example, living in a neighbourhood with lower SES may imply a disadvantaged outdoor living environment characterized by higher air pollution; lack of access to health care services, parks and green spaces, and stores with healthy food options; and lack of social support [13, 36]. For the community-level indicators of immigrant and ethnicity status, we were not able to examine the variation of CHD risk by race/ethnic group and type of immigrants. Future studies should focus on investigating the relationships between immigrant and minority status and the risk of CHD using individual-level measurement, including ethnic group and immigration status. Moreover, although all possible CHD records were obtained from the birth registry and health administrative data, it is important to note that these data are not for the sole purpose of specific research projects. Therefore, there is the potential that some CHD outcomes were misclassified. Finally, in this study, there was about 4% of the study cohort without community-level values for at least one of these SES variables due to unstable populations, absent census data, or missing maternal postal codes.

In summary, this study found that lower household income, unemployment status and living in poverty could increase the risk of CHD. Similarly, mothers with a lower education level had an increased risk of an infant with CHD. All four median household income and two education related SES indicators showed this trend and a dose response effect. Population density is not related with the risk of CHD. Immigrant and minority status could be potential protective factors, which may suggest a healthy immigrant effect. 

## Conclusions

Low maternal neighbourhood household income, poverty, lower education level, and unemployment status increase the risk of CHD, highlighting a significant social inequity in Ontario Canada. This suggests that health interventions and policies should target lower SES families. Immigration and minority status are potential protective factors, which implies a healthy immigrant effect. Further studies are required to confirm this finding by analyzing individual-level data on immigration and minority status.

## Data Availability

The data analyzed during this study is held securely at the prescribed registry BORN Ontario. Data sharing regulations prevent this data from being made available publicly due to the personal health information in the datasets. Enquiries regarding BORN data must be directed to BORN Ontario (Science@BORNOntario.ca).

## Appendices

### Appendix A

#### a) CHD diagnosis and classification of diseases in ICD-10-CA

Q20 Congenital malformations of cardiac chambers and connections.

Q21 Congenital malformations of cardiac septa (excluding patent foramen ovale in Q21.1).

Q22 Congenital malformations of pulmonary and tricuspid valves.

Q23 Congenital malformations of aortic and mitral valves.

Q24 Other congenital malformations of heart including Q24.0, Q24.2 to Q24.5, Q24.8, Q24.9,

Q25: Congenital malformations of great arteries including Q25.1 to Q25.9.

Q26 Congenital malformations of great veins, including Q26.0 to Q26.4.

#### b) Picklist values in the BIS data

**Table:**

Truncus arteriosis	
Double outlet ventricle (DOV)	
Transposition of great vessels (TGA)	
Transposition of great arteries - congenitally corrected (CCTGA)	
Double inlet ventricle (DIV)	
Single outlet ventricle	
Single ventricle (univentricular heart)	
Single ventricle / univentricular connection	
Ivemark Syndrome	
Ventricular septal defect (VSD)	
Atrial septal defect (ASD)	
Common atrium	
Atrioventricular septal defect (AVSD) (endocardial cushion defect)	
Tetralogy of Fallot (TOF)	
Aorta - pulmonary window	
Tricuspid valve dysplasia	
Valvular Anomalies	
Pulmonary (valve) atresia	
Pulmonary (valve) stenosis (PS)	
Pulmonary insufficiency	
Tricuspid atresia	
Tricuspid stenosis	
Ebstein anomaly	
Hypoplastic right heart syndrome (HRHS)	
Aortic valve stenosis	
Aortic valve insufficiency	
Mitral stenosis	
Mitral regurgitation	
Mitral valve dysplasia	
HLHS (hypoplastic left heart syndrome)	
Hypoplastic left heart syndrome (HLHS)	
Other - cardiac malformations not classified elsewhere	
Dextrocardia	
Dextrocardia	
Subaortic stenosis	
Coronary artery fistula	
Cardiomegaly	
Cardiomyopathy - dilated	
Cardiomyopathy - fetus of diabetic mother	
Cardiomyopathy - hypertrophic (HOCM)	
Diverticulum - LV	
Diverticulum - RV	
Ectopia cordis	
Pericardial/Paracardial cyst	
Pulmonary valve dysplasia	
Coarctation of aorta	
Shone’s syndrome	
Aortic arch - interrupted	
Aortic atresia	
Aortic arch - double	
Dilated ascending aorta	
Aortic arch - hypoplastic	
Right aortic arch	
Vascular ring	
Vein of Galen aneurysm	
Persistent left SVC (superior vena cava)	
Total anomalous pulmonary venous drainage (TAPVD)	
Partial anomalous pulmonary venous drainage (PAPVD)	
Bilateral SVC (superior venae cava)	
Left atrial isomerism (heterotaxy)	
Right atrial isomerism (heterotaxy)	
Scimitar syndrome	
Interrupted IVC (superior vena cava)	

### Appendix B. Sensitivity analysis

**Table 5:** Associations between different maternal SES, minority and immigration status indicators and CHD (excluding terminations and stillbirths)

Variable	Crude OR	Adjust OR
Median household income after tax		
Q1 (lowest)	1.35 (1.26–1.45)	1.29 (1.20–1.39)
Q2	1.20 (1.11–1.29)	1.15 (1.07–1.24)
Q3	1.13 (1.05–1.21)	1.09 (1.01–1.18)
Q4	1.12 (1.04–1.2)	1.11 (1.03–1.19)
Q5 (highest)	Reference	Reference
Median household income before tax		
Q1 (lowest)	1.36 (1.27–1.45)	1.31 (1.22–1.4)
Q2	1.19 (1.11–1.28)	1.15 (1.07–1.24)
Q3	1.15 (1.07–1.24)	1.12 (1.04–1.21)
Q4	1.09 (1.01–1.17)	1.09 (1.01–1.17)
Q5 (highest)	Reference	Reference
Median household income after tax adjusted by size of household		
Q1 (lowest)	1.19 (1.11–1.27)	1.20 (1.12–1.29)
Q2	1.16 (1.08–1.25)	1.16 (1.08–1.25)
Q3	1.04 (0.97–1.12)	1.04 (0.96–1.12)
Q4	1.06 (0.99–1.15)	1.06 (0.98–1.14)
Q5 (highest)	Reference	Reference
Median household income before tax adjusted by size of household		
Q1 (lowest)	1.22 (1.14–1.31)	1.23 (1.15–1.33)
Q2	1.20 (1.11–1.29)	1.19 (1.11–1.29)
Q3	1.06 (0.98–1.14)	1.05 (0.97–1.13)
Q4	1.09 (1.01–1.17)	1.09 (1.01–1.17)
Q5 (highest)	Reference	Reference
Percentage of all people with low income		
Q1 (lowest)	Reference	Reference
Q2	0.99 (0.92–1.07)	1.00 (0.93–1.07)
Q3	0.97 (0.90–1.04)	0.98 (0.91–1.05)
Q4	0.96 (0.89–1.03)	0.96 (0.90–1.04)
Q5 (highest)	1.13 (1.06–1.21)	1.13 (1.05–1.21)
Percentage of children aged 0–17 with low income		
Q1 (lowest)	Reference	Reference
Q2	0.98 (0.91–1.06)	1.00 (0.93–1.07)
Q3	0.99 (0.92–1.06)	1.02 (0.95–1.09)
Q4	1.00 (0.93–1.08)	1.02 (0.95–1.1)
Q5 (highest)	1.14 (1.06–1.22)	1.15 (1.07–1.23)
Percentage of children aged 0–5 with low income		
Q1 (lowest)	Reference	Reference
Q2	0.92 (0.86–0.99)	0.96 (0.89–1.03)
Q3	0.93 (0.88–1.00)	0.96 (0.90–1.03)
Q4	0.97 (0.91–1.04)	0.99 (0.93–1.07)
Q5 (highest)	1.16 (1.09–1.24)	1.17 (1.10–1.25)
Percentage of people aged 25–64 with a university degree or higher		
Q1 (lowest)	1.46 (1.36–1.56)	1.37 (1.27–1.48)
Q2	1.33 (1.24–1.43)	1.28 (1.19–1.38)
Q3	1.27 (1.19–1.36)	1.26 (1.17–1.35)
Q4	1.11 (1.03–1.19)	1.11 (1.04–1.19)
Q5 (highest)	Reference	Reference
Percentage of people aged 25–64 without a high school diploma		
Q1 (lowest)	Reference	Reference
Q2	1.11 (1.03–1.19)	1.11 (1.03–1.19)
Q3	1.17 (1.09–1.25)	1.15 (1.07–1.24)
Q4	1.20 (1.12–1.29)	1.18 (1.09–1.26)
Q5 (highest)	1.27 (1.18–1.36)	1.23 (1.14–1.32)
Percentage of unemployment among aged 15 or over		
Q1 (lowest)	Reference	Reference
Q2	0.94 (0.87–1.01)	0.95 (0.88–1.02)
Q3	0.99 (0.92–1.06)	1.01 (0.94–1.08)
Q4	1.02 (0.95–1.09)	1.04 (0.97–1.12)
Q5 (highest)	1.08 (1.01–1.16)	1.09 (1.01–1.17)
Percentage of employment among aged 15 or over		
Q1 (lowest)	1.22 (1.14–1.3)	1.17 (1.1–1.25)
Q2	1.1 (1.03–1.18)	1.08 (1.01–1.16)
Q3	1.04 (0.97–1.11)	1.03 (0.97–1.1)
Q4	1.10 (1.03–1.17)	1.09 (1.02–1.16)
Q5 (highest)	Reference	Reference
Percentage of minorities		
Q1 (lowest)	Reference	Reference
Q2	1.02 (0.94–1.09)	1.00 (0.92–1.08)
Q3	0.91 (0.84–0.98)	0.90 (0.83–0.97)
Q4	0.79 (0.73–0.85)	0.80 (0.74–0.87)
Q5 (highest)	0.78 (0.73–0.83)	0.84 (0.77–0.91)
Percentage of immigrants		
Q1 (lowest)	Reference	Reference
Q2	0.94 (0.87–1.01)	0.95 (0.88–1.02)
Q3	0.91 (0.85–0.98)	0.94 (0.87–1.01)
Q4	0.80 (0.75–0.86)	0.85 (0.79–0.92)
Q5 (highest)	0.74 (0.69–0.79)	0.83 (0.77–0.89)
Population density per kilometer		
Q1 (lowest)	Reference	Reference
Q2	0.96 (0.9–1.04)	1.04 (0.92–1.17)
Q3	0.98 (0.91–1.06)	1.05 (0.93–1.19)
Q4	0.95 (0.89–1.03)	1.04 (0.92–1.18)
Q5 (highest)	0.85 (0.79–0.91)	0.96 (0.85–1.08)

Note. Multivariable logistic regression models were performed to calculate adjusted ORs. Adjusted ORs for each SES variable were in one separate model adjusting for covariates including maternal age at birth, pre-pregnancy obesity, maternal smoking or social drug use or alcohol consumption, mental health illness in pre-pregnancy or during pregnancy, pre-pregnancy maternal health condition, infant sex and maternal residence in rural area

**Table 6:** Associations between SES variables, minority and immigration status and the risk of CHD (excluding terminations)

Variable	Crude OR	Adjust OR
Median household income after tax		
Q1 (lowest)	1.35 (1.26–1.45)	1.29 (1.20–1.39)
Q2	1.20 (1.11–1.29)	1.15 (1.07–1.24)
Q3	1.13 (1.05–1.21)	1.09 (1.01–1.18)
Q4	1.12 (1.04–1.20)	1.11 (1.03–1.19)
Q5 (highest)	Reference	Reference
Median household income before tax		
Q1 (lowest)	1.36 (1.27–1.45)	1.31 (1.22–1.4)
Q2	1.19 (1.11–1.28)	1.15 (1.07–1.24)
Q3	1.15 (1.07–1.24)	1.12 (1.04–1.21)
Q4	1.09 (1.01–1.17)	1.09 (1.01–1.17)
Q5 (highest)	Reference	Reference
Median household income after tax adjusted by size of household		
Q1 (lowest)	1.19 (1.11–1.27)	1.20 (1.12–1.29)
Q2	1.16 (1.08–1.25)	1.16 (1.08–1.25)
Q3	1.04 (0.97–1.12)	1.04 (0.96–1.12)
Q4	1.06 (0.99–1.15)	1.06 (0.98–1.14)
Q5 (highest)	Reference	Reference
Median household income before tax adjusted by size of household		
Q1 (lowest)	1.22 (1.14–1.31)	1.23 (1.15–1.33)
Q2	1.20 (1.11–1.29)	1.19 (1.11–1.29)
Q3	1.06 (0.98–1.14)	1.05 (0.97–1.13)
Q4	1.09 (1.01–1.17)	1.09 (1.01–1.17)
Q5 (highest)	Reference	Reference
Percentage of all people with low income		
Q1 (lowest)	Reference	Reference
Q2	0.99 (0.92–1.07)	1.00 (0.93–1.07)
Q3	0.97 (0.9–1.04)	0.98 (0.91–1.05)
Q4	0.96 (0.89–1.03)	0.96 (0.9–1.04)
Q5 (highest)	1.13 (1.06–1.21)	1.13 (1.05–1.21)
Percentage of children aged 0–17 with low income		
Q1 (lowest)	Reference	Reference
Q2	0.98 (0.91–1.06)	1.00 (0.93–1.07)
Q3	0.99 (0.92–1.06)	1.02 (0.95–1.09)
Q4	1.00 (0.93–1.08)	1.02 (0.95–1.1)
Q5 (highest)	1.14 (1.06–1.22)	1.15 (1.07–1.23)
Percentage of people aged 25–64 with a university degree or higher		
Q1 (lowest)	1.46 (1.36–1.56)	1.37 (1.27–1.48)
Q2	1.33 (1.24–1.43)	1.28 (1.19–1.38)
Q3	1.27 (1.19–1.36)	1.26 (1.17–1.35)
Q4	1.11 (1.03–1.19)	1.11 (1.04–1.19)
Q5 (highest)	Reference	Reference
Percentage of people aged 25–64 without a high school diploma		
Q1 (lowest)	Reference	Reference
Q2	1.11 (1.03–1.19)	1.11 (1.03–1.19)
Q3	1.17 (1.09–1.25)	1.15 (1.07–1.24)
Q4	1.20 (1.12–1.29)	1.18 (1.09–1.26)
Q5 (highest)	1.27 (1.18–1.36)	1.23 (1.14–1.32)
Percentage of unemployment among aged 15 or over		
Q1 (lowest)	Reference	Reference
Q2	0.94 (0.87–1.01)	0.95 (0.88–1.02)
Q3	0.99 (0.92–1.06)	1.01 (0.94–1.08)
Q4	1.02 (0.95–1.09)	1.04 (0.97–1.12)
Q5 (highest)	1.08 (1.01–1.16)	1.09 (1.01–1.17)
Percentage of employment among aged 15 or over		
Q1 (lowest)	1.22 (1.14–1.3)	1.17 (1.1–1.25)
Q2	1.10 (1.03–1.18)	1.08 (1.01–1.16)
Q3	1.04 (0.97–1.11)	1.03 (0.97–1.1)
Q4	1.10 (1.03–1.17)	1.09 (1.02–1.16)
Q5 (highest)	Reference	Reference
Percentage of minorities		
Q1 (lowest)	Reference	Reference
Q2	1.02 (0.94–1.09)	1.00 (0.92–1.08)
Q3	0.91 (0.84–0.98)	0.90 (0.83–0.97)
Q4	0.79 (0.73–0.85)	0.80 (0.74–0.87)
Q5 (highest)	0.78 (0.73–0.83)	0.84 (0.77–0.91)
Percentage of immigrants		
Q1 (lowest)	Reference	Reference
Q2	0.94 (0.87–1.01)	0.95 (0.88–1.02)
Q3	0.91 (0.85–0.98)	0.94 (0.87–1.01)
Q4	0.80 (0.75–0.86)	0.85 (0.79–0.92)
Q5 (highest)	0.74 (0.69–0.79)	0.83 (0.77–0.89)
Population density per kilometer		
Q1 (lowest)	Reference	Reference
Q2	0.96 (0.9–1.04)	1.04 (0.92–1.17)
Q3	0.98 (0.91–1.06)	1.05 (0.93–1.19)
Q4	0.95 (0.89–1.03)	1.04 (0.92–1.18)
Q5 (highest)	0.85 (0.79–0.91)	0.96 (0.85–1.08)

Note. Multivariable logistic regression models were performed to calculate adjusted ORs. Adjusted ORs for each SES variable were in one separate model adjusting for covariates including maternal age at birth, pre-pregnancy obesity, maternal smoking or social drug use or alcohol consumption, mental health illness in pre-pregnancy or during pregnancy, pre-pregnancy maternal health condition, infant sex and maternal residence in rural area

## Abbreviations

CHD: Congenital heart disease
SES: Socioeconomic status
PCCF+: Postal Code Conversion File Plus
DA: Dissemination area
BORN: Better Outcomes Registry & Network
CIHI: Canadian Institute for Health Information
DAD: Discharge Abstract Database
CIHI-DAD: The Canadian Institute for Health Information Discharge Abstract Database
CAs: Congenital anomalies
ART: Assisted reproductive technology
BIS: BORN Information System
NACRS: National Ambulatory Care Reporting System
BMI: Body mass index
AS: Antenatal specialty
PSFU: Prenatal screening follow-up
WHO: World Health Organization
OR: Odds ratio
OHIP: Ontario Health Insurance Plan
GA: Gestational age
